# Prognostic value of microRNAs in cervical carcinoma: a systematic review and meta-analysis

**DOI:** 10.18632/oncotarget.9294

**Published:** 2016-05-11

**Authors:** Shengkang Dai, Yan Lu, Ying Long, Yuehua Lai, Ping Du, Nan Ding, Desheng Yao

**Affiliations:** ^1^ Department of Gynecologic Oncology, The Affiliated Tumor Hospital of Guangxi Medical University, Nanning, Guangxi, China

**Keywords:** microRNA, prognosis, cervical carcinoma, meta-analysis

## Abstract

This systematic review is written to investigate the outcome of cervical cancer. A comprehensive search of PubMed and EMBASE was performed to identify eligible studies. Nineteen studies from thirteen articles with a total of 1,310 participants were included in this meta-analysis. Overall survival (OS), disease-free survival (DFS), and recurrence-free survival (RFS) as a prognosis for cervical cancer were extracted and calculated, if available. Pooled hazard ratios (HRs) and 95% confidence intervals (CIs) were calculated using STATA (version 12.0), resulting in the pooled HRs 0.70 (95% CI: 0.51–0.97) for OS, 1.02 (95% CI: 0.53–1.98) for DFS, and 0.56 (95% CI: 0.40–0.77) for RFS. The results indicated that cervical cancer patients with decreased microRNA expression were associated with shorter OS and RFS. It suggested that microRNAs might be promising markers for predicting the survival rate of cervical cancer.

## INTRODUCTION

Cervical carcinoma is the third most common female cancer that is closely related to human papilloma virus (HPV) infection. It was estimated that there were half a million new cases and 200, 000 deaths from cervical cancer every year [1]. Additionally, the five-year survival rate is less than 40%, especially for patients with advanced cancer [2]. Poor prognosis in cervical cancer is a chief public health problem and leads to vast hospitalization costs [3]. Although chemotherapy, radiotherapy, and related surgery have already been used as conventional treatment for cervical cancer patients, the clinical outcomes vary obviously among different patients and they are hard to be predicted. Therefore, new biomarkers which can estimate the prognosis of patients with cervical cancer are urgently required.

MicroRNAs are small noncoding RNAs, about 22–24 nucleotides long, that promote or inhibit tumor growth, progression, and metastasis [4]. They also have special expression within the tissue and are stable in the blood [5]. The expressions of various microRNAs were recently discovered to be associated with cervical cancer, indicating that they may be valuable prognostic biomarkers [6–18]. Therefore, we performed this meta-analysis to evaluate the correlation between microRNAs expression and survival rate in patients with cervical cancer.

## RESULTS

### Study characteristics

**Figure 1:**
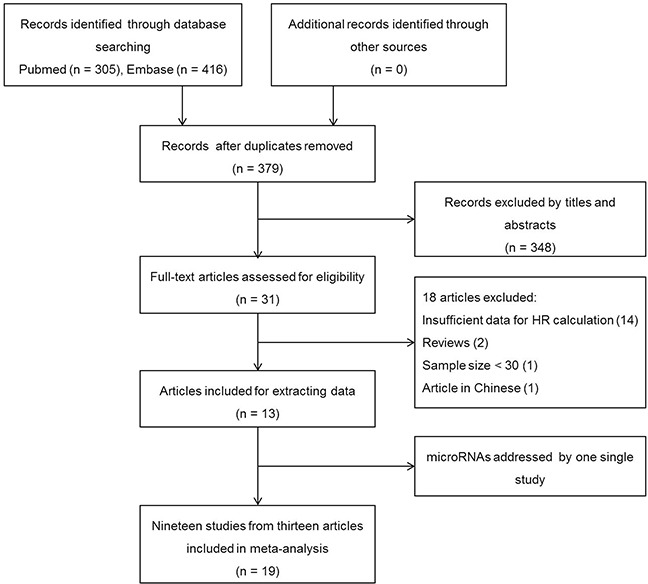
Flow diagram of the study selection process

Nineteen studies from thirteen articles with a total of 1,310 cervical cancer patients from China, Iran, and Korea were included in this meta-analysis [6–18]. These studies were all retrospective cohort studies published during 2012–2015, and they reported the prognostic value of nineteen different microRNAs in cervical cancer patients. Of the nineteen studies, eighteen reported microRNAs as a prognostic factor of OS, four discussed the association between microRNA expression and DFS, and two assessed the association of microRNA with RFS. A flow diagram of the study selection process was summarized in Figure 1. Quantitative real-time polymerase chain reaction (qRT-PCR) was used to detect microRNAs in all studies, although the cutoff values varied. The patients in the included studies were clinically staged I–IV according to the International Federation of Gynecology and Obstetrics (FIGO) staging criteria. Most patients were obtained for the studies after treatment. The low and high microRNA expression groups of each study had both lymph node metastasis and lymph node negative status. However, there was no significant difference among the included studies. The main features and extracted data of all the studies were summarized in Tables 1 and 2.

**Table 1:** The main features of enrolled studies

Author	Year	Population	Sample size	Method	Cut-off	miRNA	Survival analysis	Source of HR	Follow-up (month)
**Luo**	2015	China	88	qRT-PCR	Median	miR-26b	OS, RFS	Reported	mean 74 (5.12-98.5)
**Safari**	2015	Iran	40	qRT-PCR	Median	miR-20a	OS	DE	100
						miR-10a	OS	DE	
**Wang C**	2015	China	138	qRT-PCR	Median	miR-335	OS, RFS	Reported, DE	Mean 71(23-117)
**Wang Q**	2015	China	114	qRT-PCR	Median	miR-145	OS	Reported	median 47(11-69)
**He**	2014	China	73	qRT-PCR	ROC curve	miR-107	OS, DFS	DE	median 68.4
						miR-130a	OS, DFS	DE	
**Liang**	2014	China	335	qRT-PCR	X-tile algorithm	miR-215	DFS	Reported	60
**Ma**	2014	China	60	qRT-PCR	ROC curve	miR-205	OS	Reported	60
**Park**	2014	Korea	45	qRT-PCR	2.5-fold	miR-363-3p	OS	Reported	60
**Wang**	2014	China	54	qRT-PCR	Median	miR-31	OS	Reported	60
**Yang**	2014	China	133	qRT-PCR	Median	miR-126	OS	Reported	60
**Luo**	2013	China	60	qRT-PCR	Mean	miR-497	OS, DFS	Reported, DE	60
**Shen**	2013	China	126	qRT-PCR	Median	miR-224	OS	DE	median 51.9
**Huang**	2012	China	44	qRT-PCR	Mean	miR-125b	OS	Reported	mean 23.6 (2-70)
						miR-100	OS	Reported	
						miR-143	OS	DE	
						miR-145	OS	DE	
						miR-199a-5p	OS	DE	

HR: hazard ratio; qRT-PCR: quantitative real-time polymerase chain reaction; OS: overall survival; RFS: recurrence-free survival; DFS: disease-free survival.

**Table 2:** HRs for microRNAs

Study	miRNA	Sample size		OS		DFS		RFS		Expression associates with poor prognosis
		High level	Low level	HR (95% CI)	P	HR (95% CI)	P	HR (95% CI)	P	
**Luo 2015**	miR-26b	32	56	0.388 (0.355-0.727)	0.007	-	-	0.475 (0.311-0.573)	0.013	Low
**Safari 2015**	miR-20a	24	16	2.47 (1.31-4.66)	0.005	-	-	-	-	High
	miR-10a	24	16	2.35 (1.23-4.50)	0.01	-	-	-	-	High
**Wang C 2015**	miR-335	59	79	0.251 (0.095-0.663)	0.005	-	-	0.66 (0.47-0.92)	0.015	Low
**Wang Q 2015**	miR-145	51	63	0.63 (0.54-0.83)	0.008	-	-	-	-	Low
**He 2014**	miR-107	31	42	1.48 (0.93-2.35)	0.1005	1.89 (1.19-3.00)	0.0073	-	-	High
	miR-130a	33	40	1.38 (0.87-2.19)	0.1723	1.74 (1.10-2.77)	0.018	-	-	High
**Liang 2014**	miR-215	199	136	-	-	0.49 (0.28-0.86)	0.013	-	-	Low
**Ma 2014**	miR-205	30	30	0.33 (0.14-0.76)	0.009	-	-	-	-	Low
**Park 2014**	miR-363-3p	27	18	0.1 (0.0-0.4)	0.006	-	-	-	-	Low
**Wang 2014**	miR-31	27	27	1.482 (1.081-2.037)	0.036	-	-	-	-	High
**Yang 2014**	miR-126	71	62	0.252 (0.049-0.498)	0.003	-	-	-	-	Low
**Luo 2013**	miR-497	26	34	0.498 (0.332-0.743)	0.0167	0.64 (0.38-1.06)	0.085	-	-	Low
**Shen 2013**	miR-224	66	60	1.59 (1.12-2.26)	0.009	-	-	-	-	High
**Huang 2012**	miR-125b	4	40	0.352 (0.102-1.014)	0.057	-	-	-	-	Low
	miR-100	10	34	0.161 (0.036-0.814)	0.044	-	-	-	-	Low
	miR-143	30	14	0.55(0.29-1.04)	0.064	-	-	-	-	Low
	miR-145	26	18	0.58(0.32-1.05)	0.072	-	-	-	-	Low
	miR-199a-5p	24	20	0.56(0.31-1.01)	0.056	-	-	-	-	Low

HR: hazard ratio; 95% CI: 95% confidence interval; OS: overall survival; RFS: recurrence-free survival; DFS: disease-free survival; -: no reported.

### Correlation between microRNA expression and prognosis

**Figure 2:**
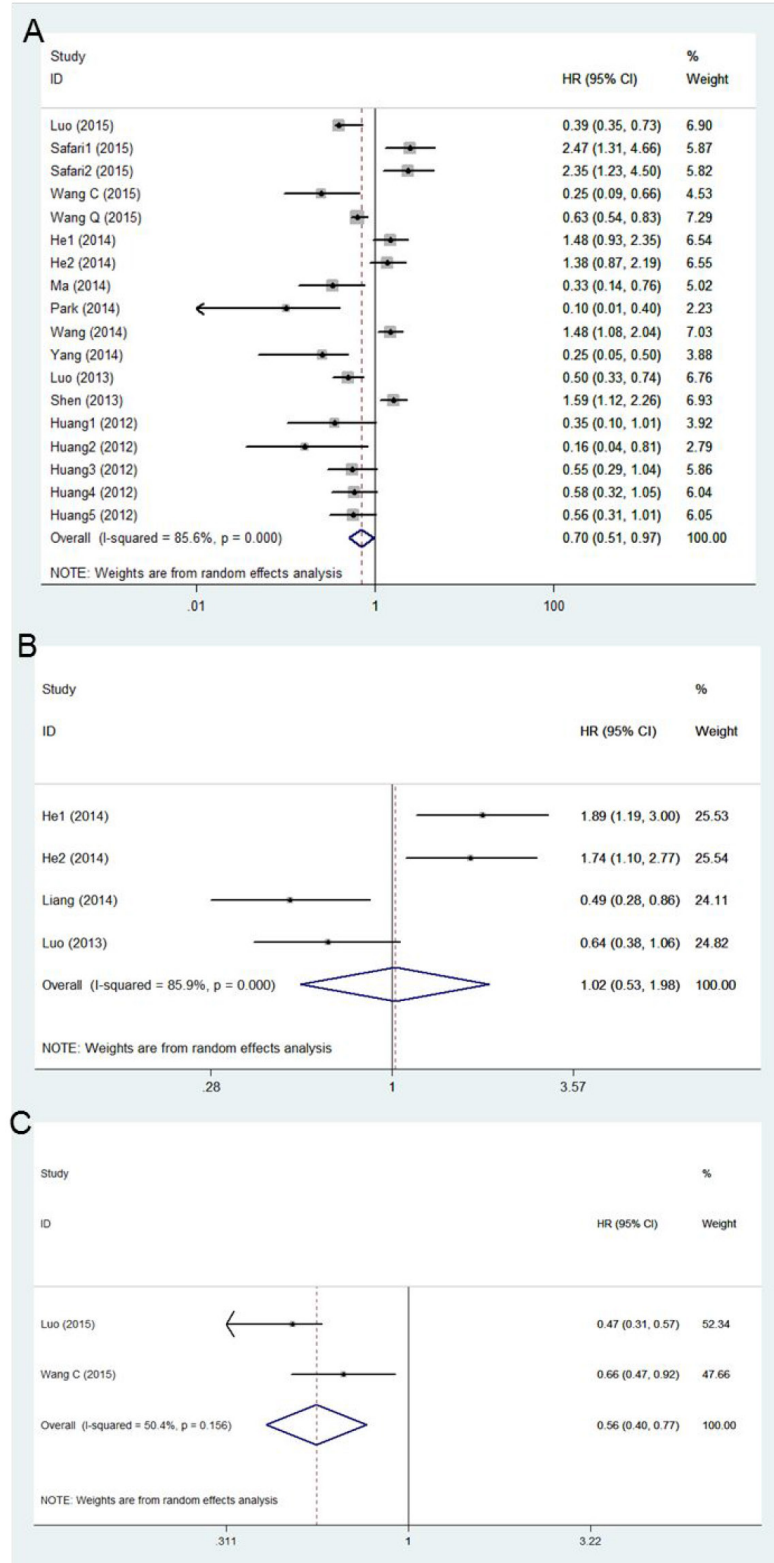
A. Forest plot of the correlation between microRNA and OS in cervical cancer patient **B.** Forest plot of the correlation between microRNA and DFS in cervical cancer patient. **C.** Forest plot of the correlation between microRNA and RFS in cervical cancer patient.

Heterogeneity was found in this meta-analysis (OS: I^2^ = 85.6%, P < 0.001; DFS: I^2^ = 85.9%, P < 0.001; RFS: I^2^ = 50.4%, P = 0.156), so the random–effects model was used to calculate the pooled HR and 95% CI values (Figure 2). According to the results displayed in Figure 2A (HR = 0.70, 95% CI: 0.51–0.97, P = 0.034) and Figure 2C (HR = 0.56, 95% CI: 0.40–0.77, P < 0.001), it was concluded that low microRNA expression indicated a poor prognosis in cervical cancer patients. However, the result displayed in Figure 2B (HR = 1.02, 95% CI: 0.53–1.98, P = 0.950) indicated that there was no obvious statistical significance.

### Meta-regression analysis

To explore the source of heterogeneity, a meta-regression analysis was performed based on five variables as shown in Table 3. For the eighteen studies on OS, sample size (coefficient = −0.0194861, P = 0.018) and cutoff value (coefficient = −0.8039943, P = 0.010) were significantly related to heterogeneity. Due to the small size of the studies on DFS and RFS, we did not perform the meta-regression analysis for these studies.

**Table 3:** Results of meta-regression on OS

Variables	Coefficient	Standard error	t	P value	95%CI
**Year**	−0.3586375	0.2829042	−1.27	0.229	−0.9750327, 0.2577577
**Country**	−0.3432079	0.4892228	−0.70	0.496	−1.409133, 0.7227171
**Sample size**	−0.0194861	0.0070848	−2.75	0.018	−0.0349225, −0.0040497
**Cut-off**	−0.8039943	0.2634475	−3.05	0.010	−1.377997, −0.22999915
**Sample type**	−1.118296	0.7370428	−1.52	0.155	−2.724175, 0.4875821

### Publication bias and sensitivity analysis

**Figure 3:**
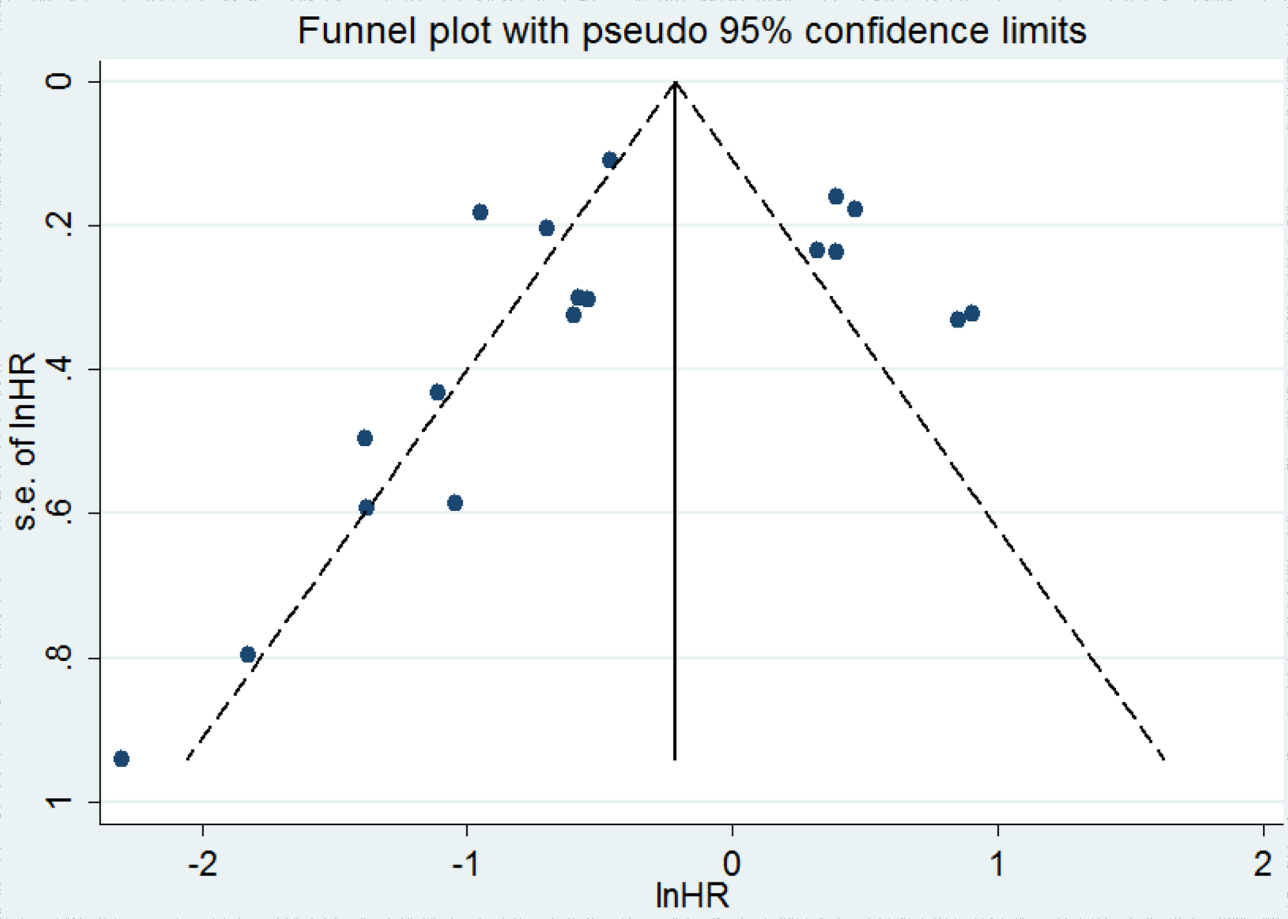
Funnel plot of eighteen studies included in this meta-analysis for OS

**Figure 4:**
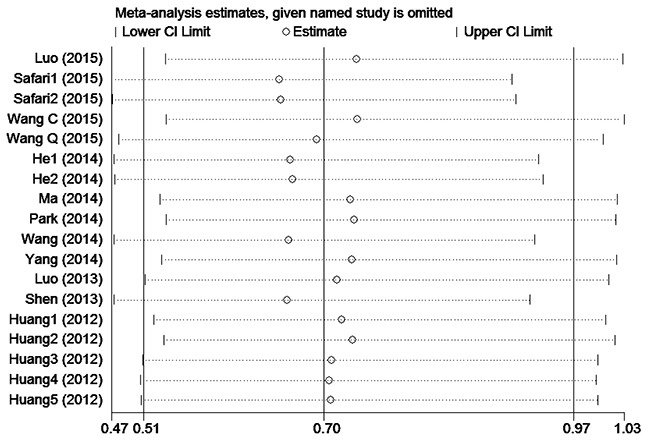
Sensitivity analysis of eighteen studies included in this meta-analysis for OS

A funnel plot and Egger's test were performed for assessing publication bias (Figure 3). The funnel plot was almost symmetrical, and the p–value of the Egger's test was 0.360 (> 0.05), indicating no obvious publication bias in this meta-analysis of OS. In order to assess whether the results were credible and stable with obvious heterogeneity, sensitivity analysis was carried out by means of omitting each study by turns (Figure 4). The result indicated that there was no obvious influence of one individual study on the pooled HR. Due to the small size of the studies on DFS and RFS, publication bias and sensitivity analysis were not performed for these studies.

## DISCUSSION

This meta-analysis is the first systematic assessment of the correlation between microRNA expression and the prognosis of patients with cervical cancer. As this meta-analysis showed, the pooled HR of OS was 0.70 (95% CI: 0.51-0.97; P = 0.034), which suggested that decreased microRNAs expression were associated with shorter OS in patients with cervical cancer. The forest plot revealed heterogeneity in this meta-analysis (I^2^ = 85.6%; P < 0.001), so we performed meta-regression analysis to explore the source. The results of the meta-regression analysis indicated that the sample size and cut-off value contributed to heterogeneity across the eighteen studies on OS. Cut-off values, country of origin, sample type, and other factors may have been sources of the heterogeneity in many similar meta-analyses [19]. We also evaluated the relationship between microRNAs expression and RFS and DFS. The pooled HR of RFS from the included studies was 0.56 (95% CI: 0.40–0.77, P < 0.001), which supported a similar conclusion. However, pooled HR of DFS from the included studies was 1.02 (95% CI: 0.53–1.98, P = 0.950). The interval of HR overlapped 1, which suggested no obvious significance.

In order to explore the source of heterogeneity in this meta-analysis, we also considered the type of cervical cancer. The main pathological classifications of cervical cancer are squamous cell carcinoma, adenocarcinoma, and adenosquamous carcinoma. In the thirteen included articles, most of them had no clear classifications, and a paper by Huang et al. revealed that the patients obtained had neuroendocrine small cell cervical carcinoma (SCCC), which is a less common type of squamous cell cervical carcinoma. After excluding this article, the pooled HR of OS was 0.80 (95% CI: 0.54–1.19, P = 0.275; I^2^ = 88.8%, P < 0.001), which suggested a higher degree of heterogeneity. The results of DFS and RFS did not change. Subgroup analysis on pathology types was not made for their undefined classifications and insufficient related information.

It is well known that a signaling pathway is a kind of enzymatic reaction pathway that can introduce extracellular signal molecules into cells through the cytomembrane. Signaling pathways cooperating with related target genes can activate or inhibit the process of cell growth, development, metabolism, apoptosis, invasion, and proliferation. For this reason, we also systematically investigated microRNAs and their potential targets and pathways included in this meta-analysis (Table 4). They can provide a reference for studying the mechanism of cervical cancer and targeted therapy.

**Table 4:** Summary of miRs with altered expression, their potential targets and pathways entered in this study

microRNA (Ref.)	Expression	Potential target	Pathway
**miR-26b (11)**	Low	USP9X, TAK1, TAB3, CDK8, PTGS2, SLC7A11	Cell growth, apoptosis, EMT and NF-κB signaling pathways
**miR-20a (12)**	High	E2F2, E2F3	Cell proliferation and modulate translation
**miR-10a (12)**	High	E2F2, E2F3	Cell invasion and metastasis
**miR-335 (13)**	Low	MERTK, Rb1, SP1, BRCA1, RUNX2, PTPRN2, TRIM29	EMT, PTEN/AKT/mTOR signaling pathways
**miR-145 (14)**	Low	p53	Cell invasion and transcription
**miR-107 (15)**	High	CCR5	Cell proliferation and invasion
**miR-130a (15)**	High	Tap63	Cell migration, invasion and metastasis
**miR-215 (16)**	Low	BRAF, KRAS, TP53, RUNX1	Cell migration, invasion and malignant progression
**miR-205 (17)**	Low	CYR61, CTGF	Cell proliferation and migration
**miR-363-3p (18)**	Low	CREB1, NOTCH1	Cell proliferation, migration and apoptosis
**miR-31 (19)**	High	ARID1A	cell proliferation, apoptosis, migration and invasion
**miR-126 (20)**	Low	EGFL7, ADAM9b, VEGF-A, CRK	VEGF/PI3K-AKT signaling pathways
**miR-497 (21)**	Low	IGF-1R	Cell growth, proliferation, migration and invasion
**miR-224 (22)**	High	RKIP	Cell metastasis, growth and proliferation
**miR-125b (23)**	Low	BAK1, ErbB2	Cell motility, invasion, glucose metabolism and chemosensitivity
**miR-100 (23)**	Low	RPSP3, PLK1, mTOR	Cell growth and migration
**miR-143 (23)**	Low	DNMT3A, KRAS, BCL-2	Cell proliferation, apoptosis and metastasis
**miR-145 (23)**	Low	BNIP3, IRS, C-MYC, YES, STAT1, MMP-11, ADAM-17	Cell proliferation, apoptosis and metastasis
**miR-199a-5p (23)**	Low	DDR1, SWI, SNF, PAK4	Cell invasion and migration

EMT: epithelial-to-mesenchymal transition; NF-κB: Nuclear factor-κB; VEGF: vascular endothelial growth factor; PI3K: phosphoinositol 3-kinase; AKT: serine/threonine kinase; PTEN: phosphatase and tensin homologue; mTOR: mammalian target of rapamycin.

This meta-analysis had several limitations that should be considered. First, significant heterogeneity existed among the studies included. Although we found that sample size and cutoff value contributed to heterogeneity, there were other potential sources. Due to the various microRNAs enrolled in this meta-analysis, the cutoff values of different microRNAs were divergent. We lacked a standard microRNA cutoff value in spite of the fact that median and mean values were often the primary cutoff value. Moreover, the normalization of the condition of qRT-PCR was also inconsistent. Second, the sample types were heterogeneous. Almost all types tested the microRNA expression in the tumor tissue, but only one was the detection of serum. As suggested in a relevant report, the synchronous detection of microRNA in the serum may conveniently provide additional information about host response and prognosis [20]. Third, part of the data derived from the relevant data extrapolation might be less credible compared with the data obtained from articles directly. Fourth, there was no obvious significance in the pooled HR of DFS that was different from the results for OS and RFS. The limited number of studies could be the possible reason. Fifth, as most of the studies enrolled were derived from Asian, potential publication bias might exist in this meta-analysis. Finally, due to the lack of related research focusing on the same microRNA, we had to calculate the pooled effect of different microRNAs for clinical evaluation. This solution was also used in other meta-analyses that lacked enough studies focusing on the same marker [19, 21]. In addition, because of certain articles without necessary data, we could not perform subgroup analysis based on microRNA category, related therapy, and other clinical characteristics, which may contribute to a portion of the heterogeneity. However, earlier related research indicated that aberrant microRNA expression was correlated with clinical characteristics such as lymph node metastasis, histological grade, and tumor diameter [22, 23].

In conclusion, we found that decreased microRNA expression was an indicator of a poor prognosis in cervical cancer patients, even if the limitations mentioned above existed. For further study, large prospective studies are needed to validate the prognostic role of microRNAs.

## MATERIALS AND METHODS

### Search strategy

Original articles investigating the prognostic role of microRNAs in cervical cancer were searched in the PubMed and EMBASE databases without time restriction. All of the articles were published before October 8, 2015. Terms such as “cervical cancer or cervical carcinoma or cervical intraepithelial neoplasia or uterine cervix cancer” and “microRNA or miRNA or miR” were jointly searched (Table 5).

**Table 5:** Search details

Electronic databases	PubMed, Embase
**Search terms # 1**	cervical cancer OR cervical carcinoma OR cervical intraepithelial neoplasia OR uterine cervix cancer
**Search terms # 2**	microRNA OR miRNA OR miR
**Search terms # 3**	Search terms # 1 AND Search terms # 2

### Selection criteria

A study was considered eligible if it met the following criteria: (1) study was written in English; (2) study investigated the prognostic value of cervical cancer patients with survival outcomes; and (3) study detected microRNA expression in tissue, serum, or plasma. An article was excluded in the following circumstances: (1) it was a review, comment, letter, or basic research article; (2) it lacked key information, such as hazard ratio (HR), 95% confidence interval (95% CI), or other useful data for extrapolating; and (3) it was a repeated study including the same samples from the same patients as a study already published. Once overlapping data were used in more than one article, the most complete study was exclusively included in this meta-analysis. Two reviewers independently evaluated the articles identified by the above criteria.

### Quality assessment

We systematically evaluated the methodological quality of all the included studies according to a critical review checklist from the Dutch Cochrane Centre proposed by the Meta-analysis of Observational Studies in Epidemiology [24]. The key points included: study population, country of the study, study design, outcome, cut-off value, microRNA detection method, and follow-up period. Each study was required to meet all of these points.

### Data extraction

The data of all eligible studies were independently extracted by two investigators. Disagreements were resolved by discussion. The following primary information was collected: name of the first author, year of publication, country of the study, sample size, disease stage, test method, cutoff value, follow-up time, and HRs of microRNA for OS, DFS, and RFS (as well as the 95% CIs and P values). If the HRs and 95% CIs were not available, we calculated them using the relevant data provided in the articles or asked for related information by emailing the authors. If only the Kaplan-Meier curves were available, we extracted the relevant data from the graphed survival plots and calculated the HRs and 95% CIs. All calculations used the methods developed by Parmar, Williamson, and Tierney [25–27].

### Statistical analysis

The pooled HRs of microRNA expression for OS, DFS, and RFS were calculated in this meta-analysis. A pooled HR < 1 means a poor prognosis for patients with low microRNA expression. In contrast, a pooled HR > 1 and a lower limit of the 95% CI of a pooled HR > 1 indicate a poor prognosis for patients with high microRNA expression [28]. Heterogeneity was assessed using a Q test and I^2^ statistic (P < 0.05 and/or I^2^ > 50% were considered statistically heterogeneous). If there was no obvious heterogeneity, a fixed-effects model was used; otherwise, the random-effects model was used. Publication bias was evaluated using the funnel plot and Egger's test (values of P > 0.05 indicated lack of publication bias) [29]. All of the analyses were performed using STATA (version 12.0). For all the results, values of P < 0.05 were considered statistically significant.
